# Effects of filtration and alpha-amylase depletion on salivary biochemical composition measurements

**DOI:** 10.1371/journal.pone.0286092

**Published:** 2023-05-26

**Authors:** Lorena Franco-Martínez, José J. Cerón, Silvia Martínez-Subiela, Asta Tvarijonaviciute

**Affiliations:** Interdisciplinary Laboratory of Clinical Analysis Interlab-UMU, Regional Campus of International Excellence Mare Nostrum, University of Murcia, Murcia, Spain; All India Institute of Medical Sciences - Patna, INDIA

## Abstract

The effects of filtration (F) and alpha-amylase depletion (AD) were assessed in n = 34 saliva samples. Each saliva sample was split into three aliquots and treated as follows: (1) no treatment; (2) 0.45μm commercial filter; and (3) 0.45μm commercial filter and affinity depletion of alpha-amylase. Then, a panel of biochemical biomarkers consisting of amylase, lipase, alanine aminotransferase (ALT), aspartate aminotransferase (AST), gamma-glutamyl transferase (GGT), alkaline phosphatase (ALP), creatine kinase (CK), calcium, phosphorus, total protein, albumin, urea, creatinine, cholesterol, triglycerides, and uric acid was measured. Differences between the different aliquots were observed in all measured analytes. The most marked changes were found in triglycerides and lipase data for filtered samples, and in alpha-amylase, uric acid, triglycerides, creatinine, and calcium results in alpha-amylase-depleted aliquots. In conclusion, the salivary filtration and amylase depletion methods employed in this report caused significant changes in saliva composition measurements. Based on these results, it would be recommended to consider the possible effects of these treatments in salivary biomarkers when filtration or amylase depletion is performed.

## Introduction

Saliva is a biofluid of increasing interest in the diagnosis and monitoring of numerous health conditions. Saliva mirrors the body’s health state and wellbeing, being 20–30% of blood proteins found in saliva, suggesting that saliva can be of clinical use in some situations [1]. Some biomarkers are found to be correlated between serum and saliva, including acute phase proteins, steroid hormones, and metabolites [2, 3]. The main advantages of saliva include that its collection is non-invasive, pain, and stress-free, and allow easier, faster, safer, and more economical collecting protocols as compared to blood [4–6]. This promotes the ease and acceptance of repeated collection from people of all ages, being of especial interest in certain situations such as epidemiological studies or when children or elderly are involved [7, 8]. Overall, saliva have a high potential as a source of non-invasive biomarkers for clinical applications [9].

Saliva also possesses a series of disadvantages for example the presence of contaminants and viscous components that can produce imprecise results [10]. The levels of particle contamination tolerated by some systems such as mass spectrometers are very limited and particle levels should be closely monitored in hydraulic systems or aircraft fuel to prevent damages. Thus, filtration of saliva samples is commonly performed prior to mass spectrometry measurements [11], as well as for exosomes identification [12] or genomics [13] analyses.

In a similar manner, the presence of high abundant proteins in saliva is known to mask potential low abundance biomarkers [14, 15]. Since alpha-amylase is the most abundant protein in human saliva, posing >50% of salivary protein content, it is commonly depleted to increase assay sensitivity in proteomic analyses [15]. The most employed method for alpha amylase depletion is the one described by Deutsch et al. (2008) [14], in which alpha amylase is depleted by affinity adsorption to potato starch, allowing the identification of 15 additional proteins not visualized before depletion using a proteomic gel-based approach.

However, these filtration and depletion of high-abundant proteins protocols may cause alterations in sample composition, such as co-depletion of several other proteins that have been described in plasma and serum [16–18]. Therefore, although it is known that saliva composition if affected by processing methods, there is a lack of information regarding the possible effects of the depletion and/or filtration protocols on the salivary biochemical composition.

The objective of this study was to evaluate if procedures such as filtration and alpha-amylase depletion could influence saliva composition. For this purpose, the effects of saliva filtration and alpha amylase depletion on a panel of different saliva components that can be used as biomarkers such as enzymes (amylase, alanine aminotransferase (ALT), aspartate aminotransferase (AST), gamma-glutamyl transferase (GGT), alkaline phosphatase (ALP), lipase, creatine kinase (CK)), inorganic elements (calcium, phosphorus), proteins (total protein, albumin) and metabolites (urea, creatinine, cholesterol, triglycerides and uric acid) was studied.

## Materials and methods

### Subjects and saliva collection

Thirty-four surplus saliva samples from a previous study [19] were employed in this study. The samples were both collected and processed for this study in 2017, and the authors did not had access to information that could identify individual participants during or after the data collection. In brief, unstimulated whole saliva samples were collected by passive drool method into a 3 mL plastic tube. The participants were asked not to drink, eat, or brush their teeth for at least an hour before providing samples. Immediately after collection, the samples were vortexed, centrifuged (3 500 g, 10 min, 4ºC) and the supernatant was stored at -80C until analysis.

All the studies were in accordance with the ethical standards of the Institutional and/or National Research Committee (1349/2016) and with the 1964 Helsinki Declaration and its later amendments or comparable ethical standards. All participants were informed of the purpose and experimental procedures of the study and signed a written informed consent form prior to their participation. This study was approved by the Ethical Committee of the University of Murcia and the Ethical Committee of the IMIB-Arrixaca.

### Saliva pre-treatments

Saliva from each subject was divided into three aliquots, which were treated as follows: (1) control, with no additional treatment (NT); (2) saliva samples were filtered through a 1.2 μm and 0.45 μm commercial filters (Millipore Corporation, Bedford, Massachusetts, USA) (F); and (3) the third aliquot was filtered through a 1.2 μm and 0.45 μm commercial filter and then passed through a homemade column with potato starch for the affinity depletion of alpha-amylase, following the protocol described by Deutsch et al. [14] (AD). The determinations were performed in duplicate.

### Biochemical measurements

All the biochemical measurements, including amylase, ALT, AST, GGT, ALP, lipase, CK, calcium, phosphorus, total protein, albumin, urea, creatinine, cholesterol, triglycerides, and uric acid, were determined using commercial kits following the manufacturer’s instructions (Beckman Coulter Inc., Fullerton, CA, USA; Olympus life and Material Science Europe GmbH, Hamburg, Germany), and were validated for its use in saliva samples. The measurements were performed using an automatic analyser (AU 600 automated biochemical analyser, Olympus, Minneapolis, Minn) using commercial assays following manufacturers’ instructions (Beckman Coulter Inc., Fullerton, CA, USA).

### Statistical analysis

Results from each biomarker and protocol were evaluated for normality of distribution using D’Agostino and Pearson omnibus normality test. As not all the data followed a normal distribution, the Friedman test followed by the Dunn’s post-test was performed to evaluate possible effect of different treatments on salivary biomarkers. This was done by comparing the results between the three groups, i.e. no treatment (NT), filtration (F) and alpha-amylase depletion (AD). The correlation was calculated using the non-parametric Spearman correlation test. A significance level of p < 0.05 was used for all analyses. Statistics were performed using GraphPad Prism 6 software (GraphPad Software Inc., La Jolla, CA, USA).

## Results

**Fig 1:**
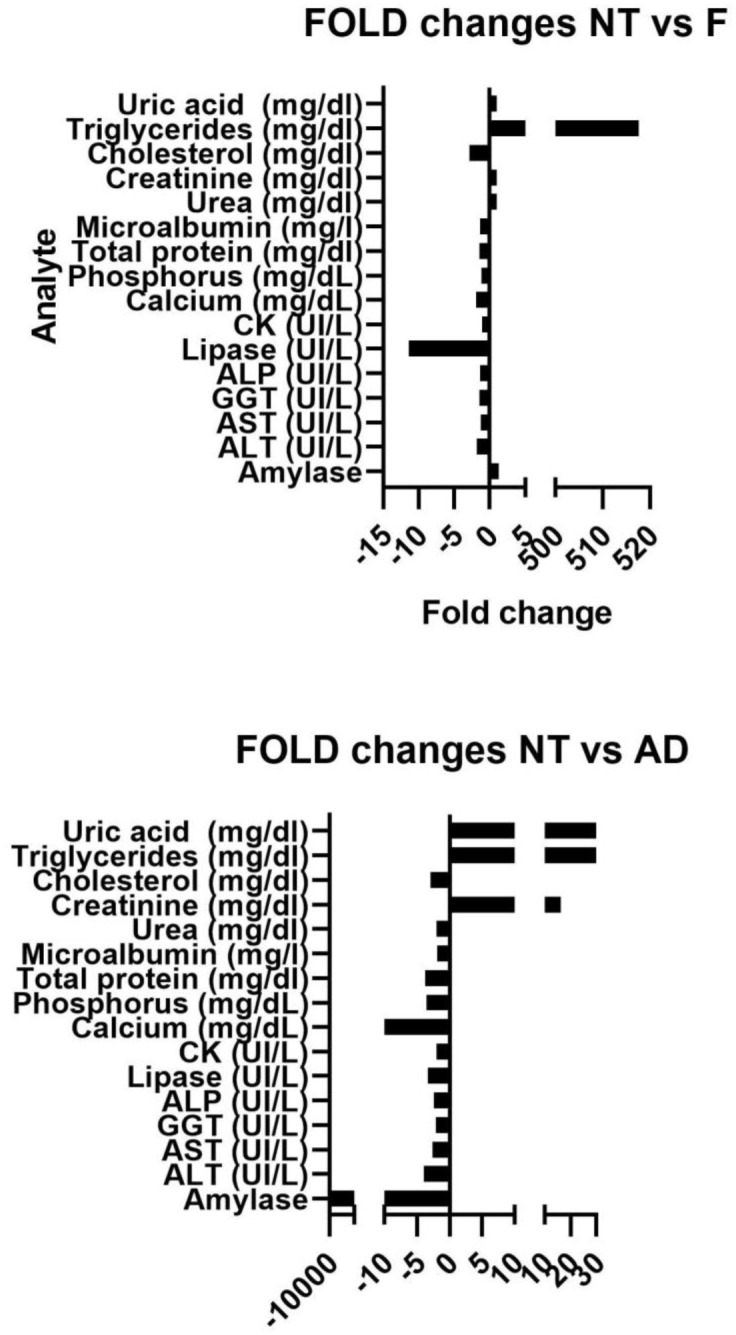
Analyte fold changes of no treatment (NP) in comparison to filtration (F, A) and amylase depletion (AD, B) measurements.

Results of the different biomarkers concentrations in saliva before and after filtration and amylase depletion are shown in Table 1 and Fig 1. All analytes measured showed statistically significant changes after filtration or amylase depletion in comparison to control group (NT). The most notable changes after filtration were observed for triglycerides (517-fold higher) and lipase (11.4-fold lower); while after AD, amylase (>11000-fold lower), calcium (49.6-fold lower), creatinine (7-fold higher), triglycerides (54.2-fold higher) and uric acid (222-fold lower) showed the most significant changes in comparison to NT.

**Table 1:** Median (25–75% interquartile range) of analytes before (NT) and after filtration (F) and filtration plus alpha-amylase depletion (AD).

		N	No treatment (NT)	Filtration (F)	Amylase- depleted (AD)	Friedman Test P value
Enzymes	Amylase (UI/mL)	26	125.4 (80.9–202)^a^	161.8 (118.6–243.5)^b^	0.01(0.0–0.02)^a,b^	<0.001
	ALT (UI/L)	34	5.9 (1.88–11.95)^a^	3.25 (1.48–7.45)^a^	1.5 (0.98–3.9)^a^	<0.001
	AST (UI/L)	34	27.65 (10.03–48)^a^	21.4 (9.63–38.2)^a^	10.4 (5.55–27.03)^a^	<0.001
	GGT (UI/L)	34	8.45 (5.98–15.45)^a^	5.9 (4.5–8.18)^a^	4 (2.98–4.95)^a^	<0.001
	ALP (UI/L)	18	7.4 (4.1–11.73)^a^	5.55 (2.83–8.7)^a^	3.5 (2.13–4.95)^a^	<0.001
	Lipase (UI/L)	34	7.95 (4.1–14.53)^a,b^	0.7 (0.7–6.43)^a^	2.4 (1.25–16)^b^	<0.001
	CK (UI/L)	34	12.45 (6.3–23.05)^a^	12.65 (8.28–18.33)^b^	6.3 (4.7–10.13)^a,b^	<0.001
Inorganic elements	Calcium (mg/dL)	34	4.96 (4.61–8.04)^a^	2.58 (2.09–4.64) ^a^	0.1 (0.03–0.1) ^a^	<0.001
	Phosphorus (mg/dL)	34	20.55 (17.08–24.3)^a^	17.92 (14.89–20.64)^a^	5.77 (3.77–8.14)^a^	<0.001
Proteins	Total protein (mg/dl)	26	151.4 (96.7–191.8)^a^	107.2 (72.96–154.4)^b^	40.66 (26.6–60.15)^a,b^	<0.001
	Albumin (mg/l)	34	3.68 (1.88–7.05)^a^	2.74 (1.88–6.47)^b^	1.91 (1.36–3.99) ^a,b^	<0.001
Metabolites	Urea (mg/dl)	34	40.65 (29.68–53.05)^a^	40.5 (29.95–50.60)^b^	20.80 (12.23–31.45) ^a,b^	<0.001
	Creatinine (mg/dl)	16	0.13 (0.10–0.19)^a^	0.13 (0.10–0.18)^b^	2.10 (1.97–5.55)^a,b^	<0.001
	Cholesterol (mg/dl)	16	1.51 (0.94–2.57)^a,b^	0.54 (0.43–0.80)^a^	0.52 (0.36–0.62)^b^	<0.001
	Triglycerides (mg/dl)	34	3.27 (1.58–5.75)^a^	1693 (1501–2188)^a^	177.2 (47.68–351.4)^a^	<0.001
	Uric acid (mg/dl)	34	2.22 (1.55–3.55)^a^	2.24 (1.69–3.64)^b^	0.01 (0.01–0.04) ^a,b^	<0.001

Same letters indicate differences of statistical relevance (Duns Test).

Correlation between the different groups were calculated for each analyte and shown in Table 2. Most enzymes showed strong positive correlations (r >0.8) between the groups. Urea and uric acid strongly correlated between NT and F (r = 0.986; r = 0.868, respectively), but not with AD. Triglycerides correlated positively between NT and AD (r = 0.39), but no between NT and F (r = -0.118, p>0.05). Calcium concentrations did not correlate between the different treatments.

**Table 2:** Correlation (r; p) between salivary concentrations before (NT) and after filtration (F) and filtration plus alpha-amylase depletion (AD) groups.

Analyte	NT vs F	NT vs AD
Amylase (UI/mL)	**r = 0.872; p = <0.001**	**r = 0.493; p = 0.003**
ALT (UI/L)	**r = 0.921; p = <0.001**	**r = 0.827; p = <0.001**
AST (UI/L)	**r = 0.959; p = <0.001**	**r = 0.912; p = <0.001**
GGT (UI/L)	**r = 0.878; p = <0.001**	**r = 0.817; p = <0.001**
ALP (UI/L)	**r = 0.930; p = <0.001**	**r = 0.812; p = <0.001**
Lipase (UI/L)	**r = 0.544; p = 0.001**	r = -0.091; p = 0.608
CK (UI/L)	**r = 0.520; p = 0.002**	**r = 0.455; p = 0.007**
Calcium (mg/dL)	r = -0.053; p = 0.765	r = -0.062; p = 0.729
Phosphorus (mg/dL)	**r = 0.733; p = <0.001**	**r = 0.448; p = 0.008**
Total protein (mg/dl)	**r = 0.807; p = <0.001**	**r = 0.775; p = <0.001**
Albumin (mg/l)	**r = 0.831; p = <0.001**	**r = 0.913; p = <0.001**
Urea (mg/dl)	**r = 0.986; p = <0.001**	r = 0.211; p = 0.231
Creatinine (mg/dl)	**r = 0.872; p = <0.001**	**r = 0.493; p = 0.003**
Cholesterol (mg/dl)	**r = 0.867; p = <0.001**	**r = 0.761; p = <0.001**
Triglycerides (mg/dl)	r = -0.182; p = 0.304	**r = 0.39; p = 0.022**
Uric acid (mg/dl)	**r = 0.868; p = <0.001**	r = 0.24; p = 0.172

Data in bold highlight statistical significance (p<0.05).

## Discussion

Saliva can be an interesting source of diagnostic biomarkers [9]; however, it is of high importance to detect the possible biases caused by its collection, processing, and storage to obtain meaningful results [20]. While much of the literature focuses on differences among different sampling methods [21–23], less emphasis is given to sample pre-processing [20]. Nevertheless, reproducible protocols for saliva pre-processing, such as removal of contaminants or high-abundance proteins, are important for precise analyses [10]. Our study provides evidence that filtration and amylase depletion, which are among the most commonly used protocols for salivary processing, caused alterations in a panel of 16 salivary analytes under the conditions of this study.

In order to reduce bias, the same aliquots of saliva were subjected to the different treatments and analysed at the same time using a high-throughput automatic analyser. The selected parameters for analysis were chosen based on: (1) its possibility of automated and fast measurement in biochemical analyses, limiting the possible artefacts due to manual analysis [24–26]; (2) their use as biomarkers to evaluate different physiological conditions and organs, including stress (amylase, lipase), liver (ALT, AST, GGT, ALP), kidney (urea, creatinine), muscle, (CK), metabolic status (cholesterol, triglycerides, albumin, total protein, calcium, phosphorus) and oxidative stress (acid uric).

Saliva filtration is a common procedure to remove contaminants such as cellular debris, turbidity and viscosity that would interfere with analytical results [20]. Therefore, it is routinely performed prior to MS analyses to avoid equipment damage, or as initial step for other techniques. Saliva filtration has been proposed as an alternative to centrifugation, reducing the need for unconventional equipment [20]. In our study, the filtration of saliva through a commercial 0.45nm filter caused alterations of statistical relevance in 9 out of 16 analytes, namely ALT, AST, GGT, ALP, lipase, calcium, phosphorus, albumin, cholesterol, and triglycerides. In all cases, except for alpha-amylase and triglycerides, lower analyte concentrations were observed after filtration. This is in concordance with previous studies in which Mini-UniPrep polyether-sulfone filters affected testosterone, de-dehydroepiandrosterone, and cortisol measurements in whole saliva [10]; while others authors did not find differences between filtered and centrifuged samples in leptin nor adiponectin concentrations [20]. In our case, these differences were more evident for triglycerides, in which the measured concentrations were increased more than 500 times after filtration in comparison to pre-treatment measurements. After filtration, samples did not show any apparent changes in terms of colour or turbidity that may interfere with the assays.

Alpha-amylase is one of the most employed salivary biomarkers of the stress response system and autonomic activity [27]. However, its high presence in human saliva can suppose a limitation to detect other markers with some techniques as for proteomics, in which high-abundance proteins can repress the signals of the lower-abundance ones [17]. Thus, many authors deplete alpha-amylase to reduce the dynamic range of proteins and unmask low-abundance proteins. This protein depletion is also performed in plasma, serum or cerebrospinal fluid, becoming a standard first step in clinical proteomics analyses aiming at biomarker discovery [28, 29]. In our study, alpha-amylase depletion using potato starch caused a marked reduction of this enzyme with concentrations close to zero (11.3 (5.52–24.25 UI/L) and >10000-fold lower concentration in comparison to non-depleted samples (NT and F) groups. The residual concentration of alpha-amylase after depletion could be caused by the saturation of the potato starch in the depletion column and has been previously reported [14]. Thus, this method allows the removal of most of the alpha-amylase content in a rapid and economic way. However, amylase-depletion changed all the others evaluated analytes in comparison to pre-treatment (NT) results. Similarly, all biomarkers, with the exception of lipase and cholesterol, differed significantly between filtration and filtration plus alpha-amylase depletion; which indicated that these changes were caused by the depletion itself and not filtration process. Therefore, cautions should be made when evaluating depleted alpha-amylase saliva samples using potato starch columns.

To determine the possible interferences that may cause filtration or alpha-amylase depletion processes, two additional tests were performed for all biomarkers using ultrapure water instead of saliva. These could also help to determine if an initial filters’ rinse could avoid these interferences by removing possible added stabilizers. Those tests were performed by quintuplicate. First, the same aliquot of ultrapure water was passed through the same filter up to five times, saving an aliquot to measure all biomarkers after each filtering process in the same run. Triglycerides increased after the first filtration from 0 to 1840 (mg/dl) and remained high for all runs. The other test consisted of five successive filtrations of new batches of ultrapure water, using the same filter. In this second test, triglycerides results increased highly after the first and second filtration (median 2171 and 289 mg/dl, respectively), and moderately from third to fifth (30.74, 14.38 and 15.85 mg/dl, respectively). Increases in lipase (as the described for saliva) were also observed in these tests with ultrapure water, while the rest of parameters results were unaltered. This, may suggests that some elements can be released from the filter during the filtration processes and interfere with triglyceride and lipase measurements, regardless of the type of sample used.

Therefore, previous rinse is not necessary for the measurement of most parameters and, in the case of triglycerides, even five consecutive rinses did not prevent the apparition of values above non-filtered samples.

Results of correlation between the three treatments varied among the different biomarkers. While some enzymes (ALT, AST, GGT, ALP) or albumin showed strong positive correlation with respect to NT after both filtration and alpha-amylase protocols; others did not correlate significantly. Therefore, the possible correlation of other biomarkers should be assessed individually; and data should be interpreted with caution because F and AD modulate biomarkers concentrations.

The present study has some limitations. First, it only evaluated the effect of one type of filter and one amylase-depletion protocol, and all samples were previously centrifuged. Thus, the use of other different materials and protocols could cause different effects on salivary composition and should be evaluated. Second, the study comprises a relatively low number of samples and does not evaluate if these changes may have clinical relevance in populations with different diseases. Therefore, future studies with larger sample size including different diseases would be desirable to determine the clinical application of our results. In addition, a panel of 16 biomarkers was used in our study and in the case of using other analytes different that those included in this report, these specific analytes should be evaluated. Nevertheless, this is a pilot study in nature and serves as a basis to indicate the influence of different sample processing protocols can have in the saliva composition.

This study demonstrates pre-processing protocols such as filtering and depleting alpha-amylase, can alter the composition of saliva samples and potentially affect the analytes of interest. Thus, cautions should be taken when interpreting results obtained through these processes. It is also important to consider the possible influence of pre-processing protocols when comparing results from different studies with varying saliva processing methods.

Overall, it is recommended that researchers using filtration or amylase depletion to consider the possible impact of these protocols to properly interpret analytes in saliva. The changes caused by these pre-processing protocols could have clinical implications in the interpretation of selected analytes, such as in triglycerides if samples have been processed differently. In conclusion, this study highlights the importance of considering potential methodological impacts on biomarker analysis in saliva.

## References

[1] Yan W, Apweiler R, Balgley BM, Boontheung P, Bundy JL, Cargile BJ, et al. Systematic comparison of the human saliva and plasma proteomes. Proteomics—Clin Appl. 2009;3: 116–134. doi: 10.1002/prca.200800140 19898684 PMC2773554

[2] Patel BJ, Dave B, Dave D, Karmakar P, Shah M, Sarvaiya B. Comparison and Correlation of Glucose Levels in Serum and Saliva of Both Diabetic and Non-diabetic Patients. J Int Oral Heal JIOH. 2015;7: 70. 26464543 PMC4588794

[3] Byrne ML, O’Brien-Simpson NM, Reynolds EC, Walsh KA, Laughton K, Waloszek JM, et al. Acute phase protein and cytokine levels in serum and saliva: A comparison of detectable levels and correlations in a depressed and healthy adolescent sample. Brain Behav Immun. 2013;34: 164–175. doi: 10.1016/j.bbi.2013.08.010 23999491

[4] Yeh C-K, Christodoulides NJ, Floriano PN, Miller CS, Ebersole JL, Weigum SE, et al. Current Development of Saliva/Oral fluid-based Diagnostics. Tex Dent J. 2010;127: 651. 20737986 PMC3742318

[5] Schulz BL, Cooper-White J, Punyadeera CK. Saliva proteome research: Current status and future outlook. Crit Rev Biotechnol. 2013;33: 246–259. doi: 10.3109/07388551.2012.687361 22612344

[6] Pfaffe T, Cooper-White J, Beyerlein P, Kostner K, Punyadeera C. Diagnostic potential of saliva: Current state and future applications. Clinical Chemistry. Clin Chem; 2011. pp. 675–687. doi: 10.1373/clinchem.2010.153767 21383043

[7] Spiesberger K, Lürzel S, Patzl M, Futschik A, Waiblinger S. The effects of play behavior, feeding, and time of day on salivary concentrations of sIgA in calves. Animals. 2019;9: 657. doi: 10.3390/ani9090657 31491913 PMC6769737

[8] G JW, K BJ, W DM. Saliva in studies of epidemiology of human disease: the UK Biobank project. Periodontol 2000. 2016;70: 184–195. doi: 10.1111/prd.12108 26662490

[9] Tvarijonaviciute A., Martinez-Subiela S., Lopez-Jornet P., Lamy E. Saliva in Health and Disease The Present and Future of a Unique Sample for Diagnosis. Tvarijonaviciute A., Martinez-Subiela S., Lopez-Jornet P., Lamy E, editor. 2020.

[10] Atkinson KR, Lo KR, Payne SR, Mitchell JS, Ingram JR. Rapid saliva processing techniques for near real-time analysis of salivary steroids and protein. J Clin Lab Anal. 2008;22: 395–402. doi: 10.1002/jcla.20281 19021269 PMC6649191

[11] Wiśniewski JR, Zougman A, Nagaraj N, Mann M. Universal sample preparation method for proteome analysis. Nat Methods. 2009;6: 359–362. doi: 10.1038/nmeth.1322 19377485

[12] Lässer C, Seyed Alikhani V, Ekström K, Eldh M, Torregrosa Paredes P, Bossios A, et al. Human saliva, plasma and breast milk exosomes contain RNA: Uptake by macrophages. J Transl Med. 2011;9: 9. doi: 10.1186/1479-5876-9-9 21235781 PMC3033821

[13] Marotz CA, Sanders JG, Zuniga C, Zaramela LS, Knight R, Zengler K. Improving saliva shotgun metagenomics by chemical host DNA depletion. Microbiome. 2018;6. doi: 10.1186/s40168-018-0426-3 29482639 PMC5827986

[14] Deutsch O, Fleissig Y, Zaks B, Krief G, Aframian DJ, Palmon A. An approach to remove alpha amylase for proteomic analysis of low abundance biomarkers in human saliva. Electrophoresis. 2008;29: 4150–4157. doi: 10.1002/elps.200800207 18937257

[15] Krief G, Deutsch O, Gariba S, Zaks B, Aframian DJ, Palmon A. Improved visualization of low abundance oral fluid proteins after triple depletion of alpha amylase, albumin and IgG. Oral Dis. 2011;17: 45–52. doi: 10.1111/j.1601-0825.2010.01700.x 20604871

[16] Granger J, Siddiqui J, Copeland S, Remick D. Albumin depletion of human plasma also removes low abundance proteins including the cytokines. Proteomics. 2005;5: 4713–4718. doi: 10.1002/pmic.200401331 16281180

[17] Fountoulakis M, Juranville JF, Jiang L, Avila D, Röder D, Jakob P, et al. Depletion of the high-abundance plasma proteins. Amino Acids. 2004;27: 249–259. doi: 10.1007/s00726-004-0141-1 15592754

[18] Stempfer R, Kubicek M, Lang IM, Christa N, Gerner C. Quantitative assessment of human serum high-abundance protein depletion. Electrophoresis. 2008;29: 4316–4323. doi: 10.1002/elps.200800211 18956433

[19] Franco-Martínez L, Tvarijonaviciute A, Martínez-Subiela S, Márquez G, Martínez Díaz N, Cugat R, et al. Changes in lactate, ferritin, and uric acid in saliva after repeated explosive effort sequences. J Sports Med Phys Fitness. 2019;59: 902–909. doi: 10.23736/S0022-4707.18.08792-3 30024129

[20] Thanakun S, Watanabe H, Thaweboon S, Izumi Y. An effective technique for the processing of saliva for the analysis of leptin and adiponectin. Peptides. 2013;47: 60–65. doi: 10.1016/j.peptides.2013.06.010 23851006

[21] Golatowski C, Gesell Salazar M, Dhople VM, Hammer E, Kocher T, Jehmlich N, et al. Comparative evaluation of saliva collection methods for proteome analysis. Clin Chim Acta. 2013;419: 42–46. doi: 10.1016/j.cca.2013.01.013 23384500

[22] Gröschl M, Köhler H, Topf HG, Rupprecht T, Rauh M. Evaluation of saliva collection devices for the analysis of steroids, peptides and therapeutic drugs. J Pharm Biomed Anal. 2008. doi: 10.1016/j.jpba.2008.01.033 18325706

[23] Mohamed R, Campbell J-L, Cooper-White J, Dimeski G, Punyadeera C. The impact of saliva collection and processing methods on CRP, IgE, and Myoglobin immunoassays. Clin Transl Med. 2012;1: 19. doi: 10.1186/2001-1326-1-19 23369566 PMC3560976

[24] González-Hernández JM, Franco L, Colomer-Poveda D, Martinez-Subiela S, Cugat R, Cerón JJ, et al. Influence of Sampling Conditions, Salivary Flow, and Total Protein Content in Uric Acid Measurements in Saliva. Antioxidants. 2019;8: 389–396. doi: 10.3390/antiox8090389 31514287 PMC6769926

[25] Dolores Contreras-Aguilar M, Escribano D, Martínez-subiela S, Martínez-Miró S, nica Rubio M, Tvarijonaviciute A, et al. Influence of the way of reporting alpha-Amylase values in saliva in different naturalistic situations: a pilot study. PLoS One. 2017;12. doi: 10.1371/journal.pone.0180100 28654668 PMC5487069

[26] Gaál Kovalčíková A, Pančíková A, Konečná B, Klamárová T, Novák B, Kovaľová E, et al. Urea and creatinine levels in saliva of patients with and without periodontitis. Eur J Oral Sci. 2019;127: 417–424. doi: 10.1111/eos.12642 31247131

[27] Bosch JA, Veerman ECI, de Geus EJ, Proctor GB. Α-Amylase As A Reliable And Convenient Measure Of Sympathetic Activity: Don’t start salivating just yet! Psychoneuroendocrinology. Psychoneuroendocrinology; 2011. pp. 449–453. doi: 10.1016/j.psyneuen.2010.12.019 21295411

[28] Silberring J, Ciborowski P. Biomarker discovery and clinical proteomics. TrAC—Trends Anal Chem. 2010;29: 128–140. doi: 10.1016/j.trac.2009.11.007 20174458 PMC2822390

[29] Gong Y, Li X, Yang B, Ying W, Li B, Zhang Y, et al. Different immunoaffinity fractionation strategies to characterize the human plasma proteome. J Proteome Res. 2006;5: 1379–1387. doi: 10.1021/pr0600024 16739989

